# Warm water treatment increased mortality risk in salmon

**DOI:** 10.1016/j.vas.2022.100265

**Published:** 2022-07-13

**Authors:** Samantha Bui, Angelico Madaro, Jonatan Nilsson, Per Gunnar Fjelldal, Martin Haugmo Iversen, Monica Fengsrud Brinchman, Birger Venås, Merete Bjørgan Schrøder, Lars Helge Stien

**Affiliations:** a Animal Welfare research group, Institute of Marine Research, Matre Research Station N-5984 Matredal, Norway; b Reproduction and Developmental Biology, Institute of Marine Research, Matre Research Station N-5984 Matredal, Norway; c Faculty of Biosciences and Aquaculture, Nord University, Postboks 1490, N-8049 Bodø, Norway; d SINTEF Ocean, Postboks 4762 Torgard, N-7465 Trondheim, Norway

**Keywords:** Warm water treatment, Delousing treatment, Mortality, Behaviour, Injuries, Stress, Atlantic salmon

## Abstract

Thermal treatment is a controversial method to control sea lice in the Atlantic salmon farming industry. This study aimed to complement the growing evidence base to document the impact of thermal treatments on salmon welfare, behaviour, physiology and health. Here, fish were treated two times (four weeks apart) for 30 s in either 27, 30, or 33 °C warm water, and parameters were compared to a procedural control (exposed to their holding temperature of 14 °C) or a negative control (where no treatments were applied). The fish had a clear behavioural response to the warm water, despite low difference between treatment and holding temperature (Δt = 13, 16 or 19 °C). Eye damages were more prevalent in the warm water treated groups than in the controls. Little difference was recorded between treatment groups in their growth and condition factor, blood plasma values, organ health, and long-term coping ability. There was, however, a significant increase in mortality as a function of temperature after the first treatment (14 °C: 6.5%, 27 °C: 5.3%, 30 °C: 12.4% and 33 °C: 18.9% mortality). The first treatment was performed only two weeks after the fish had been tagged and moved into the experimental holding tanks, while the fish had been allowed to recover for four weeks without any handling before the second treatment. The group of fish that were not subjected to any treatments (the negative control) had no mortality throughout the entire experimental period.

## Highlights

• Increased temperature gave higher mortality.
• The fish group that was not handled had no mortality.
• Eye damages were more prevalent in warm water treated groups.
• The salmon had clear behavioural reactions to the 27 °C water despite low Δt.
• The behavioural reactions were less at the second treatment compared to the first.

## 1 Introduction

The impact of the parasitic salmon louse (*Lepeophtheirus salmonis*) on the production of Atlantic salmon (*Salmo salar*) has had substantial environmental and economic costs (Solberg, 2021) . In Norway, the world's leader in salmon aquaculture, the rapid expansion of the industry is now restricted by the government due to the sustainability issues of salmon lice impacts on wild salmonid populations (Vollset et al., 2018). The industry abides by strict regulations in allowable infection levels on farmed fish, which requires acute treatments to remove lice before limits are breached. Although prevention strategies are becoming more commonly used (Barrett, Oppedal, Robinson & Dempster, 2020), they are typically not effective enough to minimise infections throughout the whole production cycle, and thus more active control measures are required; in recent years, treatments have shifted away from the use of chemotherapeutants (due to rising resistance in lice populations) towards non-medicinal approaches such as thermal bathing, mechanical treatment, or freshwater bathing, of which thermal delousing is the most common strategy (Sommerset et al., 2022; Stien, Nilsson, Sandlund & Sæle, 2022; Sviland Walde, Bang Jensen, Pettersen & Stormoen, 2021).

During thermal delousing, the salmon are pumped from their cages into a system that exposes them to heated water (28–34 °C) for up to 30 s to make the lice detach (Sommerset et al., 2022). This method is controversial: there is a rising evidence base indicating the severely negative implications for salmon welfare, however the stakes are high for the industry to have a delousing tool that is effective. The first documentation of commercial systems for thermal delousing came in 2015–2016 (Grøntvedt et al., 2015; Roth, 2016). At this time, evolution of louse resistance to the predominantly used medicinal delousing treatments (Aaen, Helgesen, Bakke, Kaur & Horsberg, 2015) meant that salmon farmers were in desperate need of new tools; as a result, the industry shifted to thermal delousing, which rapidly became the method most employed by 2017 (Overton et al., 2019). Since then, the aquaculture industry has become almost completely dependent on thermal delousing (Stien et al., 2022; Sviland Walde et al., 2021) and subsequently, thermal delousing operations is a blooming section of the industry, with multi-billion-euro investments and incomes yearly for the service boat industry (Olsen, 2020; Tveterås et al., 2020). It therefore came as a great shock when in 2019, the Norwegian Food Safety Authorities issued a warning that the method would become banned within two years, unless new evidence documenting improved welfare effects of these treatments became available. This warning was based on reports of elevated mortality (Overton et al., 2019), concern for fish welfare (Poppe, Dalum, Røislien, Nordgreen & Helgesen, 2018), and experimental studies supporting that the salmon experienced the thermal shock as painful (Nilsson et al., 2019). However, the current status (at the time of this article's publication) is that the Norwegian Food Safety Authorities issued a statement (in April 2021) – to the relief of the industry (Nygård, 2021) and to the horror of Animal Welfare NGOs (Efskind & Lybæk, 2021) – that despite high mortality and poor fish welfare still being a challenge, they will wait with their final decision until the results from ongoing research projects are finished. The present study is part of one of those projects.

Evidence shedding negative light on thermal treatments include studies showing elevated mortality after treatment (Oliveira, Dean, Qviller, Kirkeby & Bang Jensen, 2021; Overton et al., 2019; Sviland Walde et al., 2021), injured tissue after short term exposure (34–38 °C, 72–140 s; Gismervik et al., 2019), distinct aversive behavioural responses in salmon (Moltumyr et al., 2021, 2022; Nilsson et al., 2019), injuries associated with aversive behaviours during exposure (34 °C, Moltumyr et al., 2021, 2022), cardiac arrhythmias and increased blood pressure with acute increases in temperature as low as 25 °C (Clark, Sandblom, Cox, Hinch & Farrell, 2008), and increased incidence of heart injury (Poppe, Wisløff, Reed & Hofossæter, 2021). On the other hand, arguments towards positive use of thermal treatments highlight the apparent improvement in post-treatment mortality due to better equipment and more experience (Gillund & Nygaard, 2021; Stien et al., 2022; Sviland Walde et al., 2021). There has been criticisms of published results in that factors in the experiments made the conclusions incomparable to the reality of commercial practice, contending that the study by Gismervik et al. (2019) tested temperatures and durations above what is used in commercial settings, that Nilsson et al. (2019) study on behavioural responses used too small fish (∼230 g), and that Moltumyr et al. (2021; 2022) were conducted with an unrealistic difference between treatment water temperature (34 °C) and ambient water temperature (8–9 °C). For the latter point, the change in temperature (Δt) of 26 °C is much higher than the recommended Δt in the industry guidelines of 20–22.5 °C (Nygaard et al., 2020). Further arguments against the relevance of studies on physiological effects of thermal treatments included that Clark et al. (2008) study involved heating the water with the fish in it over many hours (and thereby not relevant to a 30 s treatment), and that the results from Poppe et al. (2021) are from dead fish collected after delousing and were compared with hearts from wild salmon, and therefore biased and with great uncertainty of the actual cause of death.

Thermal treatments only remove the larger, mobile stages of salmon lice (i.e., pre-adults and adults; Brunsvik, 1997; Roth, 2016). Thus, treatments allow farmers to meet the legislative thresholds of infection levels (which only use adult females for limits); however, as smaller louse stages can develop quickly in warm sea temperatures (Hamre, Bui, Oppedal, Skern-Mauritzen & Dalvin, 2019), farmed salmon risk being deloused only a few weeks apart. Moltumyr et al. (2022) tested long-term welfare effects of warm water exposure (30 s at 34 °C, Δt = 25 °C) on moderate-sized salmon (1.4 kg) three weeks apart, simulating the potential repeated treatment scenario. The authors reported that the thermal treatment resulted in significantly increased prevalence and severity of scale loss, snout wounds, various eye problems, and active fin injuries, as well as significantly reduced growth compared to procedural controls. There was no significant increase in mortality, but they reported strong behavioural responses to exposure, and that this behavioural response was the probable cause of the observed injuries (Moltumyr et al., 2022). Similarly, Moltumyr et al. (2021) observed acute negative behaviours upon exposure which contributed to poorer welfare scores immediately after treatment. In the current study, the aim was to investigate the results from Moltumyr et al. (2021, 2022) further by using larger fish (∼2 kg), a more relevant ambient temperature (14 °C), and different treatment temperatures (procedural control of 14 °C, plus 27 °C, 30 °C, and 33 °C), and thereby also different Δt's. We delved further into behavioural and physical impact of treatments by examining external welfare indicators, tissue samples of major organs, blood plasma concentrations, x-rays of the head, and monitored growth, condition factor, and mortality rates. We also included a negative control group that were reared under the same conditions, but unmanipulated throughout the study, to assess the effect of handling. In addition, five weeks after the last treatment, fish from all treatment groups were subjected to a stimulation and suppression test of hypothalamic-pituitary-interrenal axis to look for evidence of long-term effects on coping ability.

## 2 Material and methods

### 2.1 Experimental design and setup

This experiment aimed to determine the welfare and physiological effects of repeated exposure to hot water treatments, with relatively low change in temperature (Δt), on 2 kg salmon – the four treatment groups included fish exposed to 27, 30, or 33 °C thermal treatments, with a procedural control group exposed to their holding temperature 14 °C, and a negative control group that were not handled throughout the trial period. For this purpose, 600 fish were divided amongst five tanks (⌀ = 3 m, ∼5.3m^3^, 14 °C) with 120 fish in each. Four tanks held all treatment groups in a common garden (i.e. 30 fish per group per tank) and the last tank held fish that were not treated at all (negative control group).

All fish were tagged and moved into their experimental tanks 2 weeks prior to the first thermal treatment exposure (Sample 0). The planned experimental design was to expose fish to 3 sequential rounds of thermal treatment with 2 weeks between, however because of high mortality in tanks following the first treatment, the second treatment was not conducted and thus the next treatment was applied 4 weeks after the first, to give fish longer time to recover (see Results section). Thus, 2 treatments were applied to all fish (except negative controls) with a 4-week break between treatments, and a subset of ∼12 fish sampled 24 h post-treatment (at both Sample 1 and Sample 2). All fish groups, including the negative controls, were sampled 1 week after the last treatment was applied (Sample 3), and a subset of fish (30 per treatment group) were kept and redistributed equally amongst 3 new tanks to monitor for long-term post-treatment recovery. Fish were not disturbed during this subsequent 4-week period, but still monitored for mortality or abnormal behaviour. At the conclusion of this period, all fish were sampled (Sample 4). The timeline, measures taken at each sample point, and number of fish sampled are summarised in Table 1.

**Table 1:** Details of the sampling schedule and experimental activity throughout the study period (22-March to 14-June 2021), including measures recorded and number of fish sampled at each sample point.

Sample term	Date	Activity	N fish sampled per group
Sample 0 (S0) prior to start of experiment	22–23 March	Welfare scored, body size measured, PIT-tagged and moved into experimental tanks	Body size: 120 Welfare: 30
Treatment 1 (T1)	6–7 April	First thermal treatment applied	Behaviour films: 44–47
Sample 1 (S1)	7 April	Sampled 24 h post-treatment. Body size measured and blood plasma collected	7 – 12 fish in treated groups (14, 27, 30, 33 °C)
Treatment 2 (T2)	3–4 May	Second thermal treatment applied	Behaviour films: 37–49
Sample 2 (S2)	5 May	Sampled 24 h post-treatment. Body size measured and blood plasma collected. X-ray.	12 fish in treated groups (14, 27, 30, 33 °C)
Sample 3 (S3)	10–11 May	Sampled all fish groups: welfare scored, body size measured, tissue samples collected. Stimulation and suppression test of hypothalamic-pituitary-Interrenal axis. Kept subset of fish (*N* = 30 per group) for long-term monitoring	Body size: 73–89 for thermal groups, 118 for negative controls. Welfare: 30 Tissues: 6 Stress test: 11–12
Sample 4 (S4)	14 June	Final assessment, sampled all fish: welfare scored, body size measured.	28–30

### 2.2 Experimental fish

Two weeks before the first planned treatment, Atlantic salmon (AquaGen® Atlantic QTL-innOva® PRIME strain, AquaGen, Inc., Trondheim, Norway, reared at Matre Research Station, Norway) were collected from their stock tank (⌀ = 7 m, ∼58 m^3^, 8 °C), sedated (tricaine methanesulfonate, 0.1 g *L*^−1^), marked with PIT-tags (Biomark™, Idaho, USA) inserted into their stomach cavity, scored for welfare status (see section Welfare assessment below), measured for fork length (cm) and weight (g) (mean weight and SD at experiment start: 2179 *g* ± 0.41), externally tagged with coloured T-bar tags (Floy-tag Inc, Seattle, USA) to facilitate easy visual identification of their treatment group, and then transferred to their experimental holding tanks next door (S0, Table 1). Fish were systematically assigned to treatment groups in a block design (i.e., every 10 fish assigned to a group, rotating between groups). All fish were processed and transferred over two days. Throughout the experimental period, fish were fed according to a standard feed regime and held with continuous lighting. Mortality and prevalence of abnormal behaviour were monitored daily.

### 2.3 Thermal treatment application

The fish did not receive any feed the day before treatment applications, and they were also not fed during the treatment and sampling days. Thermal treatments were applied to the four tanks of treatment fish (one tank at the time), excluding the negative control group. On these days, fish were sorted by their treatment groups into separate holding vessels prior to treatment. Individual fish were gently netted (dip-net, 5 × 5 mm mesh size, ⌀ = 38 cm, depth = 42 cm, Kayoba) from their holding tank, their group identified (via the external tag colour or their PIT tag) and transferred to a holding vessel according to their treatment group (140 × 100 × 74 cm, ∼1000 L). Constant supply of seawater and oxygen was provided to the holding vessels, and water quality was monitored closely to ensure oxygen did not drop below ∼60% saturation. Once all fish in a tank were sorted, treatments began.

To apply thermal treatments, four small vessels (72 × 40 × 38 cm) for individual treatment were set up so that four fish could be treated simultaneously. Vessels were lined at the bottom with a sponge mat to add protection against mechanical damage from the plastic base (see Moltumyr et al., 2022), and half-filled with heated water (16–19 cm height) at the respective treatment temperature, which was checked for temperature and oxygen saturation (ProSolo Digital Water Quality meter, YSI, Xylem Analytics, Ohio, USA, with salinity and temperature sensor; and Handy Polary TGP, OxyGuard®, Farum, Denmark for oxygen saturation) in the stock volume immediately prior to filling the treatment vessels and also intermittently in the treatment vessels. Fish were netted from the temporary holding vessel and transferred directly to the treatment vessel for 30 s exposure, and then netted back into their original experimental holding tank. Treatment water was changed after treating 2 fish per vessel; thus, fish were treated on a rotating basis, and the same water used only for two fish before being changed. Two treatment vessels were filmed from the outside (GoPro Hero 5+, California USA) to monitor behaviour during exposure (see subsection Behavioural assessment below).

Starting with the 33 °C group, the fish were treated by this format of 2 rounds of 4 simultaneous treatments, whereby the treatment water was changed out to the temperature of the next group. Treatments cycled through the groups in this way with decreasing temperatures (i.e., 33, 30, 27, then 14 °C) repeatedly until all fish had been treated and returned to their original common garden tank. The whole process of sorting and treating 120 fish was completed in ∼1.5 h. Two tanks were processed a day, resulting in all tanks being treated over two days.

When thermal treatments were being applied, water quality was continuously monitored in the holding vessels, stock treatment water (prior to use), and treatment vessels. Temperature, oxygen saturation and total gas pressure was checked intermittently, and water quality adjusted (by adding new water or oxygen stones) if values fluctuated > ±0.4 °C beyond the treatment temperature or dropped to < 60% oxygen saturation. Total gas pressure range was always between 94 – 108% in all vessels.

### 2.4 Sampling procedure

Negative control fish were not disturbed throughout the treatments, and only treatment groups were sampled at S1 and S2 (Table 1). At these time points, temperature treatment fish were haphazardly netted from tanks to ensure even collection across groups (N given in Table 1), and immediately euthanised using an overdose of anaesthesia (tricaine methanesulfonate, 1 g *L*^−1^). Each fish was identified by their PIT-tag, then length and weight, and blood samples collected (see following subsections for details).

At S3 when all fish were processed, the holding tank water level was lowered and fish rapidly netted into a separate vessel with anaesthesia (tricaine methanesulfonate, 0.1 g *L*^−1^). Fish were identified, weighed and measured, then either immediately euthanised (tricaine methanesulfonate, 1 g *L*^−1^) and transferred to different stations depending on which samples were to be collected (i.e. for blood or tissue samples), or assessed for welfare status and moved to a new common garden holding tank (same dimensions) for long-term observation until S4. Thirty fish per group were kept for the latter goal (Table 1), with negative control fish now combined in the common garden with the other treatment groups, and a total of 3 tanks used to hold these 150 fish.

At S4, for each holding tank, the tank water level was lowered, and the fish netted (about 5 at the time) into a sedation vessel (80 × 68 × 79 cm^3^, ∼185 L) with an overdose of anaesthesia to be euthanised (tricaine methanesulfonate, 1 g *L* ^−^ ^1^). When the fish were clearly dead, they were immediately picked up from the sedation vessel, replaced with new fish for euthanasia, and body measures and welfare status recorded.

As a previous study with higher Δt and similar setup had not shown elevated mortality after treatment (Moltumyr et al., 2022), we had not set up a routine for diagnosing dead fish. However, the station veterinarian sampled four moribund fish one week after the first treatment.

### 2.5 Welfare assessment

As one of the primary study aims was to determine how acute exposure to thermal treatments affects the welfare status of fish, we monitored external indicators of welfare before and after treatments. The assessment used the FISHWELL and LAKSVEL scoring system (Nilsson et al., 2022; Noble et al., 2018), whereby indicators included skin damage (wounds, scale loss), skin bleeding, snout damage, eye damage (injury, bleeding) or opaqueness, and fin condition (caudal, dorsal, pectoral, pelvic, and anal fins). Each indicator was scored between 0 – 3, whereby 0 indicated no damage and increasing values correlated to increasing severity of condition and deviation from a healthy status.

As this study aimed to investigate whether different thermal treatments applied repeatedly affected fish welfare, we focused on deviations towards more prevalent severe cases in these indicators. Thus, the scores were used in a binary form for analyses, with the response variable representing Normal (score 0 or 1) or Severe (score 2 or 3) welfare status for each indicator.

### 2.6 Behavioural assessment

Whilst in the thermal treatment bath, 2 vessels were filmed simultaneously from the outside to observe behaviour during exposure. The camera was positioned to view the widest section of the vessels and the whole volume of water, so that films recorded salmon from a horizontal perspective. We analysed films of 44–47 individual fish in the treatment vessels of each temperature group at T1, and 37–49 individuals of each temperature group at T2. The size and volume of vessels relative to the size of fish allowed for restricted movements both horizontally and vertically. As such, behaviours during the 30 s exposure time were categorised into ‘thrashing’, ‘slow movements’, ‘standing still, and ‘loss of equilibrium’; descriptions of each behaviour are listed in Table 2. The predominant behaviour of an individual was recorded for each 5-second bin of the 30 s treatment duration, except for the first bin where the behaviour at the end of the 5-seconds was recorded to avoid registering the immediate escape behaviour from being released from the dip net into the vessel.

**Table 2:** Description of behaviours enumerated during the 30-second treatment period.

Behaviour	Description
Thrashing	Extreme and active behaviour causing excessive splashing, by jumping, wriggling, or burst swimming
Slow movements	Some tail movement, turning calmly around in the treatment vessel
Standing still	Fish appears to be standing still, keeping itself upright but no spatial movement
Loss of equilibrium	Fish is lying on its side at the bottom of the vessel

### 2.7 Tissue samples

To investigate potential damage or disease, tissues were sampled from organs at S3 whereby small sections were taken from the gills, heart, skin, muscle, kidney, brain, and both eyes. Within 2 min after anaesthesia fish tissues and organs were sampled and fixed in a 10% phosphate-buffered formalin solution for later histopathological examination. Gill samples were taken from the second gill arch. Skin and skeletal muscle samples were collected below to the dorsal fin by transverse section in the lateral line area, including both red and white muscle tissue. Kidney samples were taken from the mid-kidney. The tissue samples were stored at 4 °C until processed by an external consulting laboratory that specialises in salmon histopathological analyses (Pharmaq Analytic AS, Bergen, Norway). A general diagnostic summary was provided, and all tissue samples were scored 0 – 3 according to deviation from normal, where 0 = no specific findings, 0.5 = minimal findings, 1 = mild change, 2 = moderate change, and 3 = severe or pronounced change.

### 2.8 X-ray

At S2, all fish that were sampled (*N* = 48) were also x-rayed around the head region to inspect for internal gas accumulation (present/absent) in the eye region. Dorsal radiographs were taken of the region covering the head and the trunk according to Humborstad, Ferter, Kryvi and Fjelldal (2017) using a direct radiology system (Canon CXDI-410C Wireless, CANON, INC, Japan) and a portably x-ray unit (Hiray Plus, Model Porta 100 HF, JOB Corporation, Japan) using 88 cm distance with 40 kV and 4 mAs.

### 2.9 Physiological response

To investigate how thermal treatments influence concentrations of blood plasma parameters, we took samples 24 h after exposure to compare levels amongst groups. We focused on parameters that were unlikely to change within the short sampling time, i.e., cortisol was excluded because of the confounding influence of sampling method. Blood plasma was collected from euthanised salmon immediately after loss of consciousness, using heparinised syringes. Whole blood was placed on ice until centrifuged soon after at 5000 g for 5 min. The plasma supernatant was aliquoted and transferred into new tubes and stored at −80 °C for later analyses. From a plasma aliquot, osmolality was measured by freeze point determination in 20 μl subsamples with an Osmo Pro-Multi sample Micro Osmometer (Advanced Instruments). The concentration of the plasma *K*^+^, Na^+^, Cl^−^, Ca^++^ ions, pH and lactate glucose metabolites were analysed in 65 μl subsamples using an ABL90 FLEX blood gas analyser (Radiometer Medical ApS, Denmark). Magnesium (Mg^++^) analysis was performed using a Fluitest Mg-XB analysis kit (Biocon Diagnosemittel GmbH and Co., Germany). The kit utilizes colorimetrics by photometric absorbence analysis of xylidyl blue from a Mg-Xylidyl blue complex, which is purple in colour. 10 µL plasma for each sample was diluted individually according to kit metrics by 1 mL xylidyl blue in Eppendorf® centrifuge containers and incubated (to 26 °C) prior to deposition and analysis in 520 nm plates, controlled by Fluitest xylidyl blue (null) and Mg standard (control, total standard) and produced in mM (mmol/L).

### 2.10 Stimulation and suppression test of hypothalamic-pituitary-interrenal (HPI) axis

In order to investigate treatment impact of the on the HPI-axis feedback system, a stimulation and suppression test using adrenocorticotropic hormone (ACTH) and dexamethasone (DEX), respectively, was conducted in accordance with the previous study by Pottinger and Carrick (2001), with some minor modifications as described in Iversen and Eliassen (2014). For this test 12 fish per group, including the negative controls, were tested at S3, one week after the last treatment (T2). Briefly, fish were netted from their holding tanks, anaesthetised (tricaine methanesulfonate, 0,1 g *L*^−1^) and then injected intraperitoneally with 1 mg/kg dexamethasone (Sigma-Aldrich) in ethanol/phosphate-buffered saline (PBS) (1:3; 1 μg μL^−1^). Finally, they were transferred into 5 holding vessels (140 × 100 × 74 cm, ∼1000 L). After 24 h, the fish were anaesthetised, and 6 fish from each group were either given an intraperitoneal injection of 0.5 mL kg^−1^ adrenocorticotropic hormone (ACTH, fragment 1–24; Sigma-Aldrich) at 45 μg μL^−1^ or 0.5 mL kg^−1^ PBS. Two hours after the ACTH/PBS administration, the fish were netted, anaesthetised (tricaine methanesulfonate, 1 g *L*^−1^). Thereafter blood samples were collected and spun for plasma collection.

### 2.11 Data handling and statistical analyses

All analyses were conducted in R (R Core Team, 2019). Where applicable, residual plots were checked for assumption validation using the DHARMa package (Hartig, 2022). Non-parametric tests were used when the requirements for parametric tests were not met by the data. Mortality: Accumulated mortalities after treatments compared to negative controls were compared by Fishers's exact test for contingency tables (fisher.test, R Core Team, 2019). Since the negative control group was only in one tank, the counts for the treatment groups were combined. However, for comparison between treatments groups, it was possible to create 3-dimenisonal contingency tables (Dead[YES/NO] × Group × Tank), and thereby control for presence of any tank effect when comparing accumulated mortalities between treatment groups by Cochran-Mantel-Haenszel test (mantelhaen.test, R Core Team, 2019). Behaviour: Differences in percentage of treatment time fish exhibited the different behaviours were analysed by GLM and Tukey's multiple comparison post hoc test (glm, R Core Team, 2019), where the percentage values where arcsine transformed before analysis as recommended by Crawley (2013). Welfare indicators: Only the value of the worst scored fin, and for eye bleeding or injury, was used in analyses (i.e. one value used for Fins, and one value used for Eye condition). Differences in welfare indicator scoring between treatments at sampling points S0, S3, and S4 were compared using a Fisher's exact test for test for contingency tables, where indicators scored <2 were categorised as mild and ≥2 as moderate-severe (fisher.test, R Core Team, 2019). Histology/tissue samples: Differences in scoring of histology at S3 were tested likewise. X-ray: Differences in number of fish with possible gas behind eye between treatments were analysed by Fisher's exact test (fisher.test, R Core Team, 2019). Blood plasma concentrations: Differences in blood plasma values between temperatures and samplings were tested by GLM and Tukey's multiple comparison *post hoc* test (glm, R Core Team, 2019). Growth and condition factor: Specific growth rate was calculated as SGR = (ln(W_2_)-ln(W_1_))*T*^−1^, where W_1_ is start weight, W_2_ is end weight and T is number of days between (Brett and Groves, 1979). Condition factor (K) of individuals was calculated by: *K* = (*WL*^−3^)100, where *W* is weight (g) and *L* is fork length (cm). Change in condition factor was calculated as Δ*K* = K_2_ – K_1_, were K_1_ was start K, and K_2_ was end K. Growth and K were compared amongst thermal treatment groups only for S0, S3 and S4. Samples S1 and S2 were excluded as a) negative control fish were not sampled, and b) N was smaller and uneven at these times (Table 1). Growth and ΔK were analysed by GLM with temperature-group and start weight as factor to control for any size-driven differences in growth (glm, R Core Team, 2019). Stress test: Differences in the ACTH- and PCB-responses between treatment groups were analysed by GLM and Tukey's multiple comparison post hoc test (glm, R Core Team, 2019), while differences between tests were analysed by Kruskal-Wallis Rank Sum Test (kruskal.test, R Core Team, 2019).

### 2.12 Ethics statement

This experiment was conducted at the Institute of Marine Research's facilities in Matre, which is authorised for animal experimentation by the Norwegian Food Safety Authority (facility ID 110), and in accordance with regulations for the use of animals in experimentation (application ID: 26549).

## 3 Results

### 3.1 Mortality

**Fig. 1:**
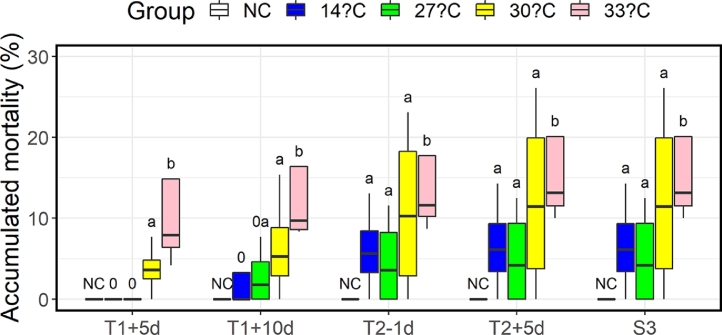
Boxplot of accumulated mortality 5 and 10 days after first treatment (T1), 1 day before second treatment (T2), 5 days after second treatment (T2) and at end of trial (S3). ‘NC’ Indicates negative control, ‘0′ indicates no significant difference (Fisher's exact test) compared to negative control (NC), while different lower-case letters indicate that the higher temperature had statistically significant different mortality than the lower (Cochran-Mantel-Haenszel Test).

Prior to the first exposure, 17 mortalities occurred, with 16 of these resulting from fish jumping out of the tanks in a single incident (lights accidentally turned on and off) one week before the first treatment (1 fish from the tank with the NC fish, and 2, 3, 5 and 5 respectively from the other four tanks). As these were unrelated to the thermal treatments, they were therefore excluded from the datasets. Mortalities include fish that were dead, or that were clearly moribund and therefore retrieved and euthanised. The mortality was high for the 33 °C group, and to lesser extent also for the 30 °C group, in the first 5 days after treatment 1 (T1+5d, Fig. 1). A veterinarian was consulted, who sampled newly dead or moribund fish (*N* = 4). The examination did not, however, reveal any obvious clinical signs of disease, and organs and tissues were therefore sent to a fish health laboratory (Pharmaq Analytic AS, Norway) for further analysis. No bacteria or viral infections were detected, nor any other disease-causing agents in these samples. The histology revealed no damages to the heart, some mild gill inflammation in three of the fish, mild deposition of calcareous material in the kidney of one fish, and three of the fish had mild to moderate damage to the eyes. Thus, there were no findings severe enough to explain the mortality. It was decided, however, to adjust the experiment and omit the next treatment which was planned two weeks after the first. Thus, the fish had four weeks to recover and only received two treatments in total, instead of three.

After the first 5 days, mortality rate gradually subsided, and the casualties changed from being fish from the 33 °C group, to fish from the lower temperatures (Fig 1). Accumulated mortality after treatment 1 stabilised across tanks at 0% for the negative control group, 6.5% for the 14 °C group, 5.3% for 27 °C group, 12.4% for the 30 °C group and 18.9% for the 33 °C group (Fig. 1). There were no mortalities after the second treatment (T2) in any of the groups (Fig. 1).

### 3.2 Behaviour in treatment vessels

**Fig. 2:**
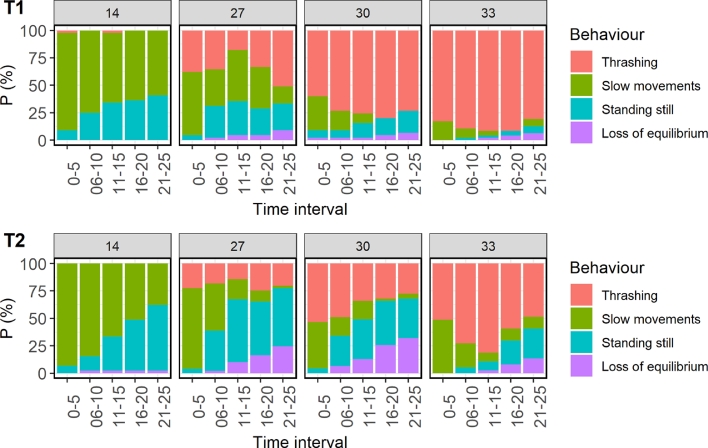
Proportion of time spent exhibiting specific behaviours in treatment vessels during first (T1) and second (T2) treatment. Time intervals during treatment are divided into the first 5 s, 6–10, 11–15, 16–20 and 21–25 s of treatment. Films of 44–47 individuals per treatment were analysed at T1, and films of 37–49 individuals at T2.

Salmon exhibited clear responses in behaviour when exposed to warm temperatures during both their first and second exposures (Fig. 2, Table 3, Table B.1). Thrashing behaviour indicated explosive movements with high intensity of splashing, jumping, bending, etc., which almost always resulted in fish colliding with the vessel walls. Incidence of thrashing and movement was notably higher in treatment groups that experienced higher temperatures, and also increased with temperature (Fig. 2, Table 3, Table B.1). Upon second exposure, thrashing behaviour subsided compared to first exposure, but the trend towards increased thrashing with temperature was still the same (Fig. 2, Table 3, Table B.1). In contrast, the procedural control (14 °C) fish exhibited almost no thrashing behaviour during either. Fish categorised as loss of equilibrium (lying on their side) typically did so soon after a period of trashing, after which they stood still momentarily before tipping sideways. This behaviour was particularly evident in the moderate temperatures (27 and 30 °C) during the second exposure (T2; Fig. 2, Table 3, Table B.1). Video clips showing examples of the behaviours are available in the online supplementary material.

**Table 3:** Mean percentage of time during treatment that the individual fish displayed the indicated behaviour at treatment 1 (T1) and 2 (T2), for each temperature exposure group. Different lower-case letters ‘abcd’ indicate significant differences between temperature treatment for the specific behaviour and treatment, while * indicates that the T2 observations are significantly different from T1 for that behaviour and group. Films of 44–47 individuals per treatment were analysed at T1, and films of 37–49 individuals at T2.

Behaviour	Treatment	14 °C		27 °C		30 °C		33 °C	
Thrashing	T1	1 ± 1%	a	35 ± 7%	b	72 ± 7%	c	87 ± 5%	d
	T2	0 ± 0%	a	20 ± 6%	b*	39 ± 7%	c*	63 ± 8%	d*
									
Slow movements	T1	70 ± 7%	a	38 ± 7%	b	12 ± 5%	c	7 ± 4%	c
	T2	67 ± 7%	a	29 ± 7%	b	17 ± 5%	c	20 ± 7%	bc*
									
Standing still	T1	29 ± 7%	a	23 ± 6%	ab	12 ± 5%	b	3 ± 3%	bc
	T2	32 ± 7%	a	40 ± 7%	a*	29 ± 7%	ab*	10 ± 5%	b*
									
Loss of equilibrium	T1	0 ± 0%	a	4 ± 3%	a	4 ± 3%	a	3 ± 2%	a
	T2	2 ± 2%	a	11 ± 4%	a	15 ± 5%	b*	5 ± 4%	a

### 3.3 Welfare assessment

**Fig. 3:**
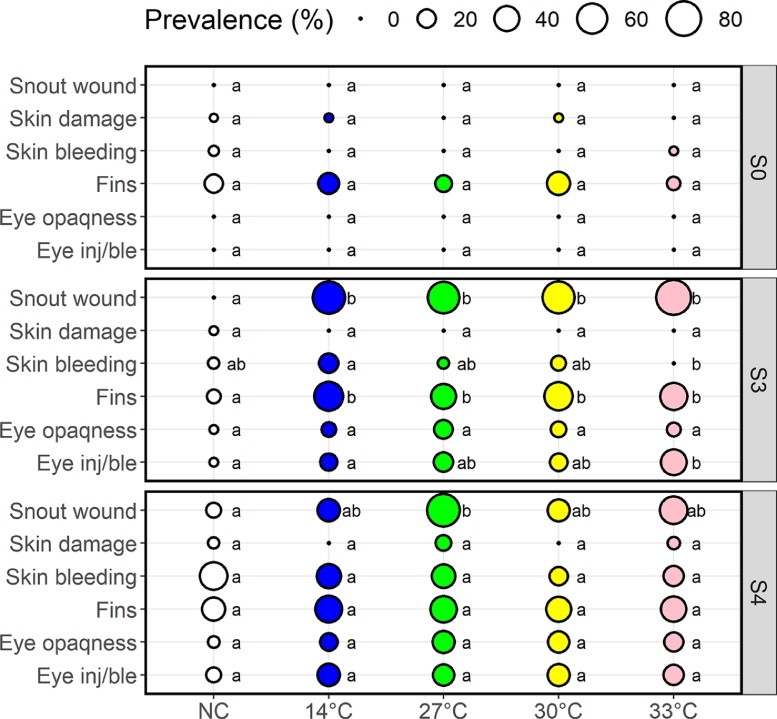
Prevalence of sampled fish amongst treatment groups that assessed as moderate-severe (score 2–3) for each welfare indicator for sampling points S0, S3 and S4. ‘Fins’ represents the worst scored fin out of the 5 individual fin indicators, and ‘Eye inj/ble’ represents the same for eye injury or bleeding. For each welfare indicator same lower-case letters indicate that there were no statistical differences in prevalence between the groups. Thirty fish were sampled from each treatment group at S0 and S3, and 28–30 fish per treatment group at S4.

Fish across all treatment groups began with similar welfare status at S0 (Fig. 3). The most prominent indicators affected were high prevalence of fish with moderate to severe fin status, with more than 10% of the sampled fish in all groups classified as having moderate-severe fin damage. It must be emphasised that all fish had worn fins prior to experiment start (S0), and the fin scores were therefore based on degree of active injury. At S3, all treatment groups had gained significant prevalence of fish with moderate to severe snout injury and/or severe fin injury compared to negative controls, but with no significant difference between treatment groups (Fig. 3), indicating that this damage likely occurred through the handling and treatment process, rather than the temperature of the water. The proportion of fish with severe eye injury increased with temperature treatment at S3, with the 33 °C group exhibiting significantly higher prevalence than the negative control and the 14 °C group, but not the 27 and 30 °C groups (Fig. 3). The results from S4 were similar to that of fish sampled at S3 for the treated fish, but with slightly less snout wounds (71 vs. 46%, *p* < 0.001). On the other hand, the subset of negative controls that had been handled when sampled at S3 and moved to new tanks together with the treated fish, had gained damage prevalence like that of the treated groups at S4 (Fig. 3).

### 3.4 X-ray

The analysis of the X-ray images at S2 revealed sign of possible gas build-up inside the eye (see Fig. A.1) in 1 of 36 thermal treated fish and none of the 12 fish sampled from the procedural control (*p* = 1.00). The affected fish belonged to the 27 °C group and was assessed to have exophthalmia on the same eye during the visual welfare assessment. The eye did not have any signs of physical injury or infection.

### 3.5 Tissue samples

**Fig. 4:**
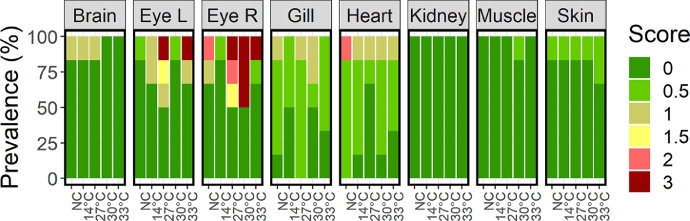
Frequency of observed scores of tissue health from histology samples taken at S3, where fish were sampled from all treatment groups (*N* = 6). Samples were taken from the brain, left and right eye (Eye L and Eye R, respectively), gills, heart, kidney, muscle and skin. Tissue samples were scored 0 – 3 according to deviation from ‘normal’ condition, where 0 = no specific findings, 0.5 = minimal findings, 1 = mild change, 2 = moderate change, and 3 = severe or pronounced change.

**Fig. 5:**
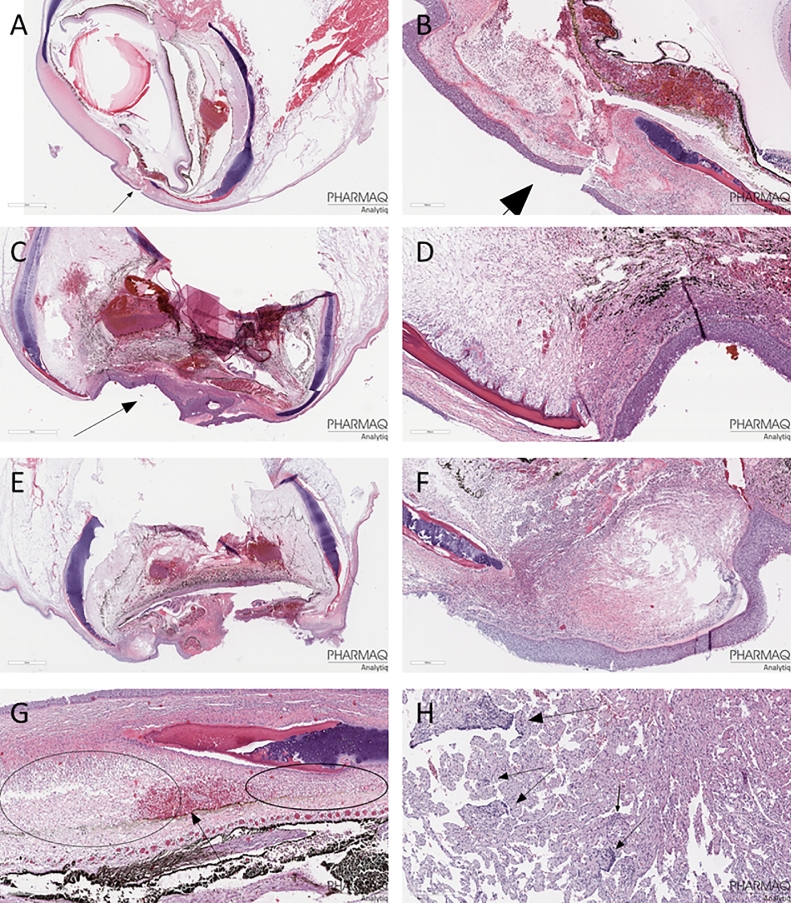
A-G: Histology examples of eye tissue with severe or pronounced change (score 3). A and B (close -up): Evidence of inflammation in cornea (keratitis). C and D (close-up): Example of keratitis and inflammation further into the eye (ophthalmitis). E and F (close-up): Example of keratitis and ophthalmitis. G: Example of bleeding, oedema and inflammation. H: Histology of heart with moderate cardiomyopathy (score 2).

In addition to the unplanned sampling of four fish during the period with high mortality, tissue and organ samples were taken from 6 fish per group at S3. The laboratory processing and analyses of these organ and tissue samples found no evidence of viral or bacterial infection. There were observed some tissue damage, but with little differences between groups (Fig. 4), except for higher prevalence of sever or pronounced change in eye tissue (score 3, bleedings, oedema and/or inflammation, see Fig. 5 for examples) in the warm water treated groups compared to the controls (*p* = 0.035). The only observation of moderate change in heart tissue was in a negative control fish (Figs. 4 and 5H).

### 3.6 Physiological response

Blood plasma parameters were measured at S1 and S2 (24 h after the first treatment and second treatment, respectively) on a subset of fish sacrificed from each temperature treatment group. There was no significant difference between treatment groups in any plasma measures at S1 or S2 (see Table B.2). For all parameters, with the exception of magnesium, values were generally lower after the second thermal exposure compared to after the first (S1 vs S2, see Table B.2).  

### 3.7 Growth and change in condition factor

The fish grew very little, or even declined in weight, from S0 to S3 (Table 4). The negative controls on average had minor growth and all the treatment groups a minor negative growth, this difference was statistically significant between the negative controls and all of the treated groups (Table 4). There was, however, no statistically significant difference between treated groups. The results where similar for ΔK. All groups (including the negative control) had lower mean K at S3 than at S0, but the decrease was greater in the treated fish and there was no significant difference between treated groups (Table 5). In comparison, from S3 to S4 the fish in all groups grew 0.2–0.4% per day and gained K (Tables 4 and 5).

**Table 4:** Average weight (g) ± SE and SGR for fish surviving till S3 and S4. Significant difference in SGR between treatment groups (GLM model corrected for start weight) is indicated by different lower-case letter. S0^3^ only includes weights of fish surviving until S3, and S3^4^ only includes weights of fish also present in sub-sample S4. Number of measurements included in each weight and SGR calculation is given in the columns N_0–3_ and N_3–4_.

Group	S0^3^	S3	SGR_0–3_	N_0–3_	S3^4^	S4	SGR_3–4_	N_3–4_
NC	2245 ± 37	2381 ± 45	+ 0.089 ± 0.02 a	116	2505 ± 82	2885 ± 91	0.403 ± 0.06 a	29
14 °C	2100 ± 40	2055 ± 44	÷ 0.040 ± 0.02 b	85	2101 ± 80	2378 ± 82	0.364 ± 0.05 a	30
27 °C	2138 ± 41	2111 ± 49	÷ 0.033 ± 0.02 b	87	2315 ± 87	2516 ± 90	0.244 ± 0.07 a	28
30 °C	2207 ± 39	2162 ± 42	÷ 0.039 ± 0.02 b	83	2263 ± 65	2681 ± 77	0.374 ± 0.06 a	30
33 °C	2131 ± 55	2033 ± 54	÷ 0.081 ± 0.02 b	72	2240 ± 87	2529 ± 110	0.330 ± 0.06 a	29

**Table 5:** Average condition factor (*K* ± SE) and ΔK for fish surviving until S3 and S4. A significant difference in ΔK between treatment groups (GLM model corrected for weight) is indicated by *. S0^3^ only includes weights of fish surviving until S3, and S3^4^ only includes K's of fish also present in sub-sample S4. See Tabl4 4 for N.

Group	S0^3^	S3	ΔK_0–3_	S3^4^	S4	ΔK_3–4_
NC	1.19 ± 0.01	1.13 ± 0.01	÷ 0.067 ± 0.015a	1.13 ± 0.02	1.17 ± 0.02	0.036 ± 0.036a
14 °C	1.17 ± 0.01	1.07 ± 0.01	÷ 0.095 ± 0.010b	1.06 ± 0.02	1.13 ± 0.02	0.059 ± 0.013a
27 °C	1.17 ± 0.01	1.08 ± 0.01	÷ 0.086 ± 0.009b	1.11 ± 0.01	1.18 ± 0.06	0.021 ± 0.019a
30 °C	1.18 ± 0.01	1.08 ± 0.01	÷ 0.101 ± 0.009b	1.08 ± 0.02	1.15 ± 0.02	0.047 ± 0.016a
33 °C	1.16 ± 0.01	1.07 ± 0.01	÷ 0.093 ± 0.010b	1.07 ± 0.02	1.11 ± 0.04	0.047 ± 0.038a

### 3.8 Stimulation and suppression test

**Fig. 6:**
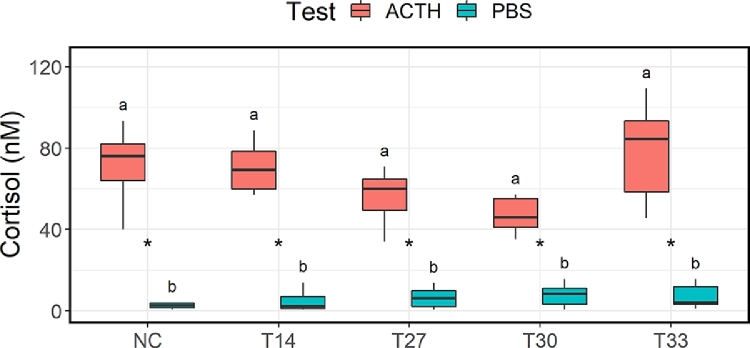
Boxplot of cortisol response in salmon that receive adrenocorticotropic hormone (ACTH) and salmon that had received phosphate-saline solution (PBS). All fish were injected 24 h previously with dexamethasone . The stimulation and suppression test was done at sampling point S3, i.e., one week after the last treatment (T2). * indicates a significant difference between the ACTH and PBS cortisol response, while identical lower-case letters indicate no statistical difference between the response of different treatment groups.

For all treatment groups, there were significant differences in cortisol response between the fish that had received ACTH inoculation and the fish that had received PBS (Fig. 6). There was, however, no differences in cortisol response between any of the treatment groups, for neither the ACTH- nor the PBS-fish (Fig. 6).

## 4 Discussion

The results from this study indicate little difference between Atlantic salmon experiencing short-term exposure to 27, 30, or 33 °C water with regards to their growth, general welfare status and physiological response, however warmer temperatures did induce stronger behavioural responses, higher mortality rates and higher prevalence of eye injury. The handling and treatment procedure negatively influenced growth during the experimental period compared to fish that were not handled and also led to reduced condition factor at S3 compared to at S0. Similar to previous studies (Moltumyr et al., 2021, 2022), we observed that fish exposed to warm water thrashed in the treatment vessel, while this behaviour was almost completely absent in the procedural control fish (14 °C). Thrashing can thus be said to be a clear behavioural response salmon have to warm water. We also observed that the proportion of time fish exhibited thrashing behaviours increased from 27 to 30 °C, and further from 30 to 33 °C. This is similar to Nilsson et al. (2019) who found a clear behavioural response in Atlantic salmon (∼230 g) exposed to 28 °C, compared to salmon exposed to lower temperatures, with a further increase in behavioural response when the salmon were exposed to water temperatures above 34 °C. Ashley, Sneddon and McCrohan (2007) showed that rainbow trout (*Oncorhynchus mykiss*) has mechanothermal nociceptors in the skin with a thermal threshold around 29 °C and polymodal nociceptors with a thermal threshold around 33 °C. These thresholds are however averages, and vary greatly between individuals (Ashley et al., 2007). It is therefore to be expected that in a given population, some individuals will not have the same strong behavioural response to 27 °C as others, and similarly that at 33 °C more individuals will thrash throughout the entire exposure than at 30 °C.

An important difference between the current and previous studies (Moltumyr et al., 2021, 2022; Nilsson et al., 2019) is that starting temperature was 14 °C in the current vs. 8–9 °C in the previous studies. That the fish, likewise as in these earlier studies, exhibited thrashing at 27 °C, despite significant lesser difference in ambient vs. treatment temperature (i.e. Δt), supports that there is a heat threshold for salmon pain perception around 27–28 °C independent of starting temperature. This is also supported by Peterson and Anderson (2011) who found that Atlantic salmon smolt acclimatised to 18 °C had a clear behavioural response when the water in the tank was heated to 27 °C, and not when heated to 24 °C.

The predominant behaviour in procedural control fish were ‘slow movements’, which was not considered a strong response; this represents what is commonly observed when a salmon is suddenly confined in a smaller volume of water whereby there are some initial escape or exploratory behaviours, followed by slow swimming or ceased movements. In these experimental fish, ‘standing still’ could be interpreted as them settling down (as perhaps occurred in 14 °C fish) or a coping behaviour occurring while still highly stressed. Freezing and hiding is a well-known passive defence strategy in fish (Øverli et al., 2007) and it is also known that some fish with poor welfare will exhibit passive responses to stressful stimuli, such as freezing (Huntingford et al., 2006). This, together with that the ‘standing still’ behaviour typically was superseded by ‘loss of equilibrium’ in the fish exposed to warm water support that these fish, were not settling down, but on the contrary, highly stressed.

Fish lying on their side is likely a negative behaviour corresponding to loss of equilibrium and possibly lack of coping to the stressor (Gismervik et al., 2019; Nilsson et al., 2019). In Nilsson et al. (2019) the fish reached this endpoint of equilibrium loss after ∼100 s at 36–38 °C and samples showed severe tissue damage in the gills and brain Gismervik et al. (2019). In our case, however, the exposure time was only 30 s, the temperature ≤33 °C, and the fish woke up and reacted when netted out of the exposure vessel. Possible reasons for why loss of equilibrium occurred so early in the current study, compared to after ∼100 s in Nilsson et al. (2019), may be added stress from not having been netted directly from the holding tank, but via a holding vessel, their larger body size, added confinement stress from being in a small treatment vessel, added (head) injury from thrashing towards the vessel walls, or possibly difference in health and welfare status of the fish.

Interestingly, the thrashing behaviour was typically maintained throughout the exposure duration during the first treatment but was less dominating during the second treatment. This might be due to habituation; it is well known that salmon can habituate to a frightening and/or painful stimuli (Fernö et al., 2020). The number of exposures is, however, normally far higher than one for salmon to exhibit significant habituation (Bratland et al., 2010; Madaro et al., 2016). On the other hand, the stressor in the current study, i.e. confinement in warm water, is probably stronger than the stressors in these habituation studies (flashing lights, chasing). Another possible explanation is that the fish at the second exposure had less energy available to perform thrashing behaviour than in the first. This explanation is supported by the loss of weight in treated fish between S0 and S3.

The treatment groups lost up to 4.6% of their body weight over the ∼50 days including the two treatment applications (S0–S3 period) compared to the slight growth of negative control fish that did not undergo any procedures. This was also reflected in the condition factor of treatment fish, which also was reduced during this period, suggesting that the stress load of repetitive handling and treatment affected appetite and/or feed conversion. The reduction in welfare status after treatments could also represent a chronic stressor that would further supress appetite. Moltumyr et al. (2022) similarly recorded lower growth rates after repeated 34 °C treatment, however no difference in condition factor between thermal-treated and control fish. Little data is available from commercial settings on the appetite of salmon after large-scale thermal delousing events, however anecdotal evidence indicates poor growth after delousing but is largely associated with other factors (e.g., physical damage and reduced welfare, high mortality rates; (Sommerset et al., 2020).

The thrashing behaviour during thermal exposure has previously been linked to increased incidence of external injuries (Moltumyr et al., 2021, 2022), however in the current study, these differences between the procedural control and the thermal treated groups were probably partly masked by the procedure, whereby fish first had to be netted from their tank to a temporary holding vessel, then to the treatment vessel and after 30 s back again to their original tank. That handling can have a negative effect on salmon is underlined by that the handling procedure in the current experiment resulted in 6.5% mortality in the procedural control vs. no mortality in the fish that were left alone (the negative control). The risk of handling is further underlined by that after the negative controls had been handled when sampled at S3 and moved to new tanks together with the treated fish, they had gained damage prevalence at S4 like that of the treated groups. Handling is an intrinsic part of thermal delousing, also in commercial settings, so the damage we observed here is not necessarily exclusive to the experimental setting. In a commercial setting the fish will not be netted by dip nets, but they will be crowded in large numbers, pumped into the treatments system, and transported and pushed through various pumps, slides, and chambers where they risk harming themselves towards each other and the various physical structures of the system in question. Although both operators of delousing systems and fish farmers seek to limit the risk to the fish, fish health professionals report high incidence of damage to fins, eyes, gills, and scale loss and skin bleeding after both thermal and mechanical delousing in the industry (Sommerset et al., 2020, 2022).

Both the welfare scores and the histology analyses showed increased prevalence of eye injury in thermal treated fish compared to controls. Gismervik et al. (2019) also found increased incidence of eye injury, albeit after both higher temperatures (34–38 °C) and longer exposure times (72–140 s). Moltumyr et al. (2021) found that 3 of 39 salmon treated at 34 °C for 30 s had severe eye injuries vs. 0 of 19 of the procedural controls, and Moltumyr et al. (2022) reported that 12% of the salmon had severe eye injuries two weeks after the last thermal treatment compared to 0% in the procedural controls. Cumulatively, this suggests that warm water itself may make the eyes of salmon more vulnerable to injury, and not necessarily only a consequence of mechanical damage due to acute stress behaviour. This hypothesis needs to be further investigated.

One important note for discussion is the impact of treatments on tissues sampled after commercial thermal treatments compared to our tank study; specifically, Poppe et al. (2018) and 2021) and Østevik et al. (2022) linked damage to the gills, the skin and heart to thermal treatments which contrasts with the results of the present trial. There are many possible explanations for this discrepancy. Firstly, both Poppe et al. (2018)) and (2021) are based on samples sent to their lab from the industry after thermal delousing and may therefore be assumed to be predominantly cases where the delousing for some unknown reason has gone wrong. Secondly, the changes in heart morphology presented in Poppe et al. (2021) were found in both mechanical and thermal treated fish, and the comparison was heart morphology in wild salmon. Deviations in heart morphological resulting from intensive smolt production (Frisk et al., 2020) could explain some of this difference. Thirdly, Østevik et al. (2022) found increased damaged in gill tissue after both thermal and mechanical delousing, suggesting that also other parts of the delousing process may play a part, for example the pumping of the fish from the sea cage into the treatment system. This demonstrates the importance of performing controlled laboratory trials, where it is possible to keep all other components of the handling identical, except the one component of main interest (here, the warm water), however controlled trials also have their weaknesses. The most prominent are that the fish will usually all be from the same background and sample populations will be relatively small. For instance, injuries that happen in only one of every 1000 fish in a commercial cage (containing 50 000–200 00 fish) will likely be missed and would be almost impossible to get statistically significant in a small-scale lab experiment. However, this one-in-a-thousand fish might be exactly the one outlying individual that a health professional in the field would send for analysis to laboratories such as in Poppe et al. (2021). The single fish found with possible gas build up inside the eye in the X-ray images may be an example of this. It is therefore important to underline that absence of significantly different results between treatments in the current study only mean that we were not able to demonstrate an effect, not that such an effect does not exist.

There were no differences in blood ions (Na^+^, Cl^−^, Ca^++^, *K*^+^and Mg ^++^), pH, osmolarity, glucose, or lactate concentration 24 h post-treatment, neither after the first nor the second treatment. This supports the theory that the stress caused by the procedure itself is likely masking the effects of the exposure to different temperatures. However, the plasma parameters measured at S2 were significantly different to the ones measured at S1, and more similar to typical control values (Noble et al., 2018). This observation suggests that fish could had perceived the first treatment as more severe than the second, possible due to habituation (see above) or insufficient tank acclimatation before T1, thus resulting in a higher change of plasma parameters, or in a longer recovery time than 24 h, to return to pre-stress levels.

Possible reasons for why mortalities in thermal treated groups were elevated after the first treatment but not the second include the potential habituation of individuals to the experience, leading to lower stress responses and less subsequent injuries. This is supported by Schreck, Jonsson, Feist and Reno (1995) who achieved lower mortality during transportation compared to controls for Chinook salmon (*Oncorhynchus tshawytscha*) that had been crowded and then fed (as a positive reward) twice every day for 6 days before the transportation event. However, both this study, and the before mentioned studies on habituation in Atlantic salmon(Bratland et al., 2010; Madaro et al., 2016) required far more than one exposure to achieve the habitation effect. It is therefore possible that the change in behaviour seen from T1 to T2 is a result of the fish having less energy too struggle and thereby harm themselves, as supported by their loss in weight between S0 and S3. However, since 40% of the fish exposed to 33 °C thrashed throughout the entire exposure period also at T2 (see Table B.1) there should also be mortalities after T2 if this was the only explanation. Another possibility is that the ‘weak’, compromised fish were culled from the group after T1 and only the stronger fish remained at T2. However, in the latter case, there should still be weak individuals left in the thermal 27 °C group at T2, as this group had significantly less mortality than the 33 °C group after T1. A more compelling explanation is that at the first treatment, there had been only two weeks since the fish had been moved from the stock tank into the smaller experimental tanks (⌀ = 7 m vs. 3 m) and underwent internal and external tagging. Moving fish, moving fish into smaller tanks and tagging are all known health risks, especially if the fish are not given sufficient time to recover (Espmark et al., 2017; Vollset et al., 2020). It is likely that this, compromised the fish at the first treatment, while the zero mortality after the second treatment supports that they at this time had recovered from the tagging. That tagged salmon subjected to a stressor may have considerable increased risk for mortality is exemplified in Wright et al., 2019, where less than 1% of the untagged salmon died, while almost 40% of the tagged salmon died in the modified sea cages (snorkel sea cages), and all the tagged salmon in the standard sea cages survived. That the fish, were compromised, due to insufficient recovery from tagging, is further supported by that there was only 3% mortality after thermal treatment at 34 °C in Moltumyr et al. (2022) compared to 18.9% after thermal treatment at 33 °C in the current study. Moltumyr et al. (2022) had salmon of similar size and also a similar experimental procedure, but here the salmon were allowed to recover for 5 weeks before they underwent the first treatment compared to only 2 weeks in the current study. That health status is important for the mortality outcome after thermal treatment is also in line with data from the industry. Overton et al. (2019), did for instance find higher increase in mortality after thermal delousing in fish populations that already had elevated mortality (compromised) compared to fish populations with low mortality (uncompromised).

The results of the suppression and stimulation test did not reveal any differences between any of the treatment groups. Dexamethasone is a potent synthetic corticosteroid, with high affinity for the glucocorticoid receptors. In healthy fish, as for cortisol, dexamethasone exerts negative feedback on both the hypothalamus and the anterior pituitary of the HPI axis, thus inhibiting the secretion of CRH and ACTH respectively, preventing the inter-renal activation and thus the cortisol release. On the other hand, healthy fish suppressed with dexamethasone first and then stimulated with ACTH will mount a cortisol response. In the current experiment, the administration of dexamethasone caused a similar suppression for the stress axis and cortisol release for all treatment groups, and the groups also mounted similar cortisol response when injected with ACTH. These data suggest that at the end of the trial, fish were able to cope with the applied stress and that the previous treatments did not seriously compromise the stress coping capacity of the fish long term (Iversen & Eliassen, 2014).

Combined with previous knowledge, the results from this study indicate that it is the absolute treatment temperature, rather than the experienced change in temperature, that is the significant factor that determines how much the salmon responds behaviourally to the warm treatment water. Combined with previous results, it is also clear that salmon eyes are at risk with thermal treatments, either directly, or indirectly via their thrashing response, or a combination where the warm water weakens the eyes while the thrashing behaviour leads to the actual damage. There was significant increase in mortality as a function of temperature after the first thermal exposure, but not after the second when the fish had been allowed to recover; this indicates that higher temperature treatment incurs a higher risk for the salmon, but also that there may possibly be a habituation effect, or more likely that the salmon at the first treatment had not recovered sufficiently from the internal tagging. That the health status of the fish is important is known from industry data. Future research focus should therefore concentrate on finding “health markers” that can be used by the farmers and the fish health professionals, to determine whether a fish population is suitable for thermal delousing or not.

## Ethical statement

This experiment was conducted at the Institute of Marine Research's facilities in Matre, which is authorised for animal experimentation by the Norwegian Food Safety Authority (facility ID 110), and in accordance with regulations for the use of animals in experimentation (application ID: 26549).

## Declaration of Competing Interest

The authors declare the following financial interests/personal relationships which may be considered as potential competing interests: Lars Helge Stien reports financial support was provided by The Norwegian Seafood Research Fund.

## Appendix Supplementary materials

Image, application 1

Image, video 2

Image, video 3

Image, video 4

Image, video 5
