# Supplementary material for: Warm water treatment increased mortality risk in salmon
Source: Vet Anim Sci. 2022 Jul 13;17:100265. doi: 10.1016/j.vas.2022.100265 (PMC9309499; doi:10.1016/j.vas.2022.100265)
Supplement: Supplementary file 1 [file mmc1.pdf]

A. Appendix. Supplementary figures

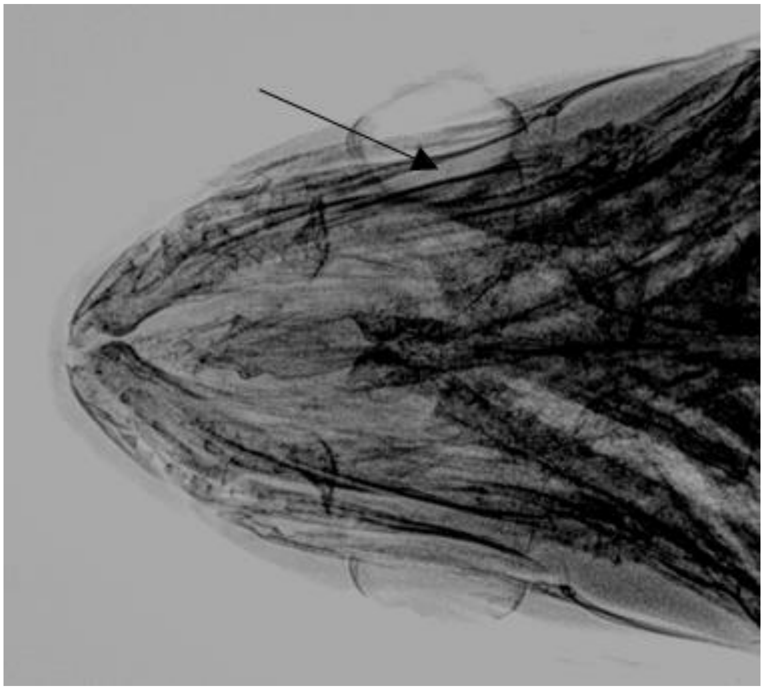

Fig. A.1. Dorsal radiograph showing build-up of gas inside the eye of one fish belonging to the 27 °C group. The whole eye has a less radio-dense appearance indicating gas (arrowhead). The other eye is normal.

B. Appendix. Supplementary tables

Table B.1. Percentage of fish in each treatment group trashing 0 to 1/5 (0 to 20 %), 2/5 to 3/5 (40 to 60 %), 4/5 to 5/5 (80 to 100 %) of the exposure time at treatment 1 (T1) and treatment 2 (T2).

**Percentage of time**	**Treatment**	**14 °C**	**27 °C**	**30 °C**	**33 °C**
0 to 20 %	T1	100 %	56 %	9 %	0 %
	T2	100 %	74 %	46 %	14 %
40 to 60 %	T1	0 %	27 %	31 %	19 %
	T2	0 %	16 %	32 %	46 %
80 to 100 %	T1	0 %	18 %	60 %	81 %
	T2	0 %	10 %	21 %	40 %

Table B.2. Mean percentage of measured blood plasma values at 24 hours after first treatment (S1) and second treatment (S2). There were no significant differences between treatments between any temperatures at neither S1 nor S2. P-values for test for differences between S1 and S2 are given in the last row

					**S1: 24 hours after treatment 1 (T1)**					
Group (°C)	N	pH	Na$^+$ (mmol/L)	Cl$^-$ (mmol/L)	Ca^{++} (mmol/L)	K$^+$ (mmol/L)	Glucose (mmol/L)	Lactate (mmol/L)	Osmolality (mOsm)	Mg^{++} (mmol/L)
14	12	6.91 ± 0.05	174 ± 1.2	149 ± 1.4	1.11 ± 0.05	3.61 ± 0.08	5.34 ± 0.21	2.82 ± 0.16	361± 7.6	1.03 ± 0.08
27	9	6.96 ± 0.05	178 ± 3.6	152 ± 3.4	1.06 ± 0.04	3.66 ± 0.08	5.82 ± 0.56	2.97 ± 0.21	369 ± 6.2	0.86 ± 0.06
30	8	6.95 ± 0.04	177 ± 2.4	151 ± 2.3	1.10 ± 0.05	3.62 ± 0.14	5.82 ± 0.24	2.90 ± 0.23	358 ± 4.7	0.90 ± 0.06
33	8	6.91 ± 0.04	173 ± 2.1	146 ± 1.7	1.00 ± 0.03	3.59 ± 0.13	5.39 ± 0.21	2.79 ± 0.27	345 ± 5.1	0.95 ± 0.05
					**S2: 24 hours after treatment 2 (T2)**					
Group (°C)	N	pH	Na$^+$ (mmol/L)	Cl$^-$ (mmol/L)	Ca^{++} (mmol/L)	K$^+$ (mmol/L)	Glucose (mmol/L)	Lactate (mmol/L)	Osmolality (mOsm)	Mg^{++} (mmol/L)
14	12	7.10 ± 0.06	169 ± 0.7	142 ± 0.6	0.99 ± 0.03	3.19 ± 0.06	4.91 ± 0.13	2.19 ± 0.17	350 ± 3.9	1.17 ± 0.19
27	12	7.08 ± 0.07	171 ± 1.0	144 ± 0.8	0.89 ± 0.02	3.10 ± 0.12	4.82 ± 0.18	2.12 ± 0.25	348 ± 3.8	0.92 ± 0.04
30	12	7.08 ± 0.04	169 ± 0.7	142 ± 0.8	0.96 ± 0.02	3.07 ± 0.12	4.97 ± 0.15	2.01 ± 0.13	341 ± 2.7	1.04 ± 0.05
33	12	7.05 ± 0.06	169 ± 0.9	142 ± 1.3	0.91 ± 0.02	3.06 ± 0.08	5.02 ± 0.16	2.27 ± 0.15	347 ± 3.0	0.94 ± 0.02
					**S1 vs S2**					
P (S1 vs S2)		<0.001	<0.001	<0.001	<0.001	<0.001	<0.001	<0.001	<0.001	0.273

C. Appendix. Supplementary videos

Video clips showing examples of the behaviours are available in the online supplementary material.

Supplementary video 1: Video of salmon being exposed to 14 °C.

Supplementary video 2: Video of salmon being exposed to 27 °C.

Supplementary video 3: Video of salmon being exposed to 30 °C.

Supplementary video 4: Video of salmon being exposed to 33 °C.
